# Movement disorders following mechanical thrombectomy resulting in ischemic lesions of the basal ganglia: An emerging clinical entity

**DOI:** 10.1111/ene.16219

**Published:** 2024-02-01

**Authors:** Leonardo Rigon, Danilo Genovese, Carla Piano, Valerio Brunetti, Valeria Guglielmi, Angelo Tiziano Cimmino, Irene Scala, Salvatore Citro, Anna Rita Bentivoglio, Eleonora Rollo, Riccardo Di Iorio, Aldobrando Broccolini, Roberta Morosetti, Mauro Monforte, Giovanni Frisullo, Pietro Caliandro, Alessandro Pedicelli, Anselmo Caricato, Giovanna Masone, Paolo Calabresi, Giacomo Della Marca

**Affiliations:** ^1^ Dipartimento di Neuroscienze Università Cattolica del Sacro Cuore Rome Italy; ^2^ Dipartimento di neuroscienze, Organi di Senso e Torace Fondazione Policlinico Universitario A. Gemelli IRCCS – UOC Neurologia Rome Italy; ^3^ The Marlene and Paolo Fresco Institute for Parkinson's Disease and Movement Disorders New York University Langone Health New York New York USA; ^4^ UOC Radiologia e Neuroradiologia, Dipartimento di diagnostica per immagini, radioterapia oncologica ed ematologia Fondazione Policlinico Universitario A. Gemelli IRCCS Roma Italy; ^5^ Neuro Intensive Care Unit, Fondazione Policlinico Universitario Agostino Gemelli IRCCS Università Cattolica del Sacro Cuore Rome Italy

**Keywords:** acute ischemic stroke, basal ganglia, mechanical thrombectomy, post‐stroke movement disorders

## Abstract

**Background and purpose:**

Post‐stroke movement disorders (PMDs) following ischemic lesions of the basal ganglia (BG) are a known entity, but data regarding their incidence are lacking. Ischemic strokes secondary to proximal middle cerebral artery (MCA) occlusion treated with thrombectomy represent a model of selective damage to the BG. The aim of this study was to assess the prevalence and features of movement disorders after selective BG ischemia in patients with successfully reperfused acute ischemic stroke (AIS).

**Methods:**

We enrolled 64 consecutive subjects with AIS due to proximal MCA occlusion treated with thrombectomy. Patients were clinically evaluated by a movement disorders specialist for PMDs onset at baseline, and after 6 and 12 months.

**Results:**

None of the patients showed an identifiable movement disorder in the subacute phase of the stroke. At 6 and 12 months, respectively, 7/25 (28%) and 7/13 (53.8%) evaluated patients developed PMDs. The clinical spectrum of PMDs encompassed parkinsonism, dystonia and chorea, either isolated or combined. In most patients, symptoms were contralateral to the lesion, although a subset of patients presented with bilateral involvement and prominent axial signs.

**Conclusion:**

Post‐stroke movement disorders are not uncommon in long‐term follow‐up of successfully reperfused AIS. Follow‐up conducted by a multidisciplinary team is strongly advisable in patients with selective lesions of the BG after AIS, even if asymptomatic at discharge.

## INTRODUCTION

Movement disorders secondary to acute ischemic stroke (AIS) of the basal ganglia (BG) often occur with variable latency [1] and present with a variety of symptoms [2, 3, 4, 5]. Both hyperkinetic and hypokinetic post‐stroke movement disorders (PMDs) can be observed in patients following ischemic injury of the BG, owing to their key role in motor function control [1, 3, 6, 7, 8, 9]. The deep nuclei more frequently linked to the secondary onset of PMDs are the striatum, globus pallidus and thalamus [1, 9, 10]. However, no clear correlation between the site of ischemia and the subsequent PMD clinical phenotype has yet been reported [6]. Different phenotypes of PMDs seem to manifest with different latency after the acute event, with chorea being the earliest (hours to days after the acute event), and dystonia the latest (up to several years later) [1, 7, 11]. A prominent feature of PMDs is the asymmetrical distribution, usually contralateral to the BG ischemic lesion, even though bilateral impairment can also be observed [2].

The overall outcome of AIS due to large vessel occlusion (LVO) of the anterior intracerebral circulation has been dramatically changed by the advent of mechanical thrombectomy (MT), either alone or following intravenous thrombolysis (IVT) [12]. The clinical efficacy of MT is strongly dependent on the level of recanalization of the occluded vessel [13].

The BG are particularly susceptible to ischemic injury due to their anatomical and metabolic characteristics [14]. The vascular supply of the BG is usually guaranteed by terminal branches of the M1 segment of the MCA and of the anterior cerebral artery, respectively, the lenticulo‐striate arteries and the recurrent artery of Heubner [15]. The lack of collateral circulation exposes these structures to an increased risk of ischemic injury. In addition, the BG present several peculiar cellular and subcellular characteristics, such as the propensity to glutamate‐mediated excitotoxicity exacerbated by hypoperfusion [14] and a high oxidative metabolism, which make them more vulnerable to ischemic damage as compared to the neighboring white matter structures. Consistent with this, occlusion of the M1 portion of the MCA, even when promptly and successfully treated with MT, often leads to a lesion of these deep nuclei and represents an anatomical lesional model involving the BG [16]. Indeed, as previously described [17, 18], M1 occlusions almost inevitably induce a lenticulo‐striate infarction, regardless of the efficacy and timing of the MT, whereas the neighboring deep white matter might be spared with prompt intervention [19].

Longitudinal data on PMDs following relatively selective BG ischemic lesions in patients treated with MT are lacking. The aim of this pilot prospective study was to assess the prevalence and the clinical features of PMDs secondary to AIS due to M1 occlusion successfully treated with MT in the short and long term.

## MATERIALS AND METHODS

In this single‐center, longitudinal prospective study, we considered consecutive patients treated with MT between August 2020 and December 2022, meeting the following inclusion and exclusion criteria. The study was approved by the local ethics committee (*Comitato Etico Fondazione Policlinico Universitario Agostino Gemelli* IRCSS, protocol number 5137).

### Inclusion criteria

The inclusion criteria were: AIS due to occlusion of the M1 segment of the MCA, either alone or with concomitant occlusion of the homolateral internal carotid artery; substantial radiological recanalization following treatment with MT as defined by modified Thrombolysis In Cerebral Infarction (mTICI [20]) score ≥ 2B; radiological evidence of recent BG ischemic lesion; age ≥ 18 years; and signed specific informed consent.

### Exclusion criteria

Exclusion criteria were extensive cortical and subcortical ischemic lesions (defined by involvement of more than a third of the MCA vascular supply territory [21]) and history of movement disorders or severe cognitive decline.

### Treatment of AIS

Patients were selected for revascularization therapies (IVT and/or MT) according to the current guidelines [22, 23, 24, 25]. MT was performed in eligible patients either as primary treatment or after bridging with IVT. The efficacy of MT was radiologically established according to the mTICI score [20]. Procedures achieving mTICI score ≥2B (anterograde reperfusion of more than half of the treated artery ischemic territory) were considered successful treatments [13, 26].

### Assessments

All patients were screened for family history of movement disorders and previous exposure to dopamine receptor blocking agents (DRBAs). Presence of major risk factors for AIS, namely, history of previous ischemic stroke, diabetes, hypertension, dyslipidemia and tobacco smoking, was assessed at baseline (Table S4). In the acute phase, clinical features regarding the AIS were collected: National Institutes of Health Stroke Scale (NIHSS) score [27] at onset and at discharge, the pre‐event and discharge modified Rankin scale (mRS) score [28], the mTICI score, eventual bridge treatment with IVT, and stroke subtype according to TOAST (Trial of ORG 10172 in acute stroke treatment) classification [29]. All patients underwent neuroradiological examination (either brain computed tomography or magnetic resonance imaging [MRI]) while hospitalized, as per clinical practice. Images from the acute phase (within 4 days from the stroke) were analyzed to assess lesion extension and the involved structures.

Patients who were able to collaborate were screened in the acute phase for movement disorders and cognitive impairment with a thorough neurological assessment and evaluation with the Unified Parkinson's Disease Rating Scale [30] (UPDRS) and the Montreal Cognitive Assessment [31] (MoCA); in case of evidence of a PMD different from parkinsonism, specific scales were used: the Fahn, Tolosa, Marin scale [32], the Burke Fahn Marsden Dystonia Rating Scale [33] and the Abnormal Involuntary Movement Scale [34] for tremor, dystonia and other hyperkinetic movements, respectively. The presence of subtle motor signs, such as mirror movements (MMs) or reduced arm swing (RAS) was established clinically. Patients were successively evaluated by video‐taped full neurological examination and the UPDRS score at 6 and 12 months to assess the possible onset of PMDs; other scales were used according to the clinical presentation. Cognition was tested using the MoCA scale at the 6‐ and 12‐month follow‐up visits.

According to the current definition [35], parkinsonism is a clinical syndrome characterized by the presence of bradykinesia plus at least one of rest tremor and rigidity. In our study, we considered clinically relevant a score ≥2 on the UPDRS III with items as follows: items 3–7 for tremor, items 10–14 for rigidity and items 15–22 for bradykinesia. In the absence of obvious confounders (e.g., severe hemiparesis or spasticity), we confirmed the clinically established diagnosis of parkinsonism in patients presenting with UPDRS scores ≥2 for at least one item evaluating bradykinesia + a UPDRS score ≥2 in at least one item evaluating tremor and/or rigidity. Other movement disorders were established clinically and evaluated with the most appropriate scale. UPDRS score was further subdivided into: axial subscore (sum of items 23–28) and items assessing appendicular tremor, rigidity and bradykinesia (items 20–26), dichotomized into homolateral and contralateral to the ischemic lesion subscores.

The overall clinical outcome of the AIS was assessed via telephone call using the mRS 3 months after the event [36].

Scores obtained in the pre‐ and post‐event and the 3‐month mRS assessments were dichotomized per global clinical independence into 0–2 versus 3–6 [37].

When the follow‐up visit could not be scheduled due to COVID‐19 pandemic‐related restrictions or severe clinical condition (mRS score >4), clinical evaluations were postponed until the next follow‐up window.

### Statistical analysis

To assess the risk factors for PMD development, between‐group differences in demographic and clinical variables were analyzed using the Mann–Whitney *U*‐test or the Wilcoxon signed rank test for continuous variables and Pearson's chi‐squared test or Fisher's exact test for categorical variables, as applicable.

All statistical computations were two‐tailed, and a *p* value <0.05 was considered significant. Statistical analyses were performed using XLSTAT software, version 2021.3.1 (Addinsoft, Inc., Brooklyn, NY, USA).

## RESULTS

### Baseline assessment

**FIGURE 1:**
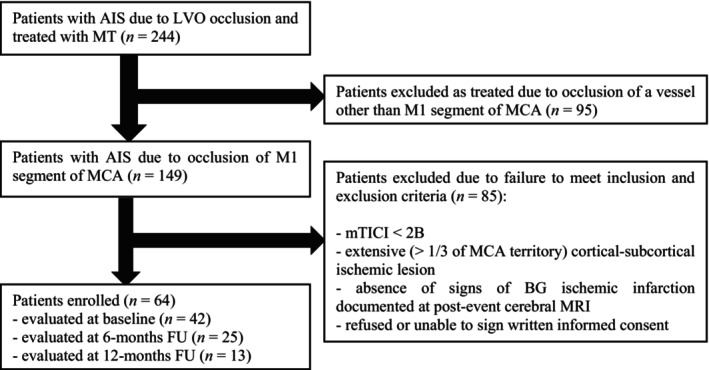
Flowchart of the study. AIS, acute ischemic stroke; BG, basal ganglia; FU, follow‐up; LVO, large vessel occlusion; MT, mechanical thrombectomy; MCA, middle cerebral artery; mTICI, modified treatment in cerebral ischemia score; MRI, magnetic resonance imaging.

Between August 2020 and December 2022, 244 consecutive patients were treated with MT for AIS due to LVO at our center. Among them, 149 presented with an occlusion of the M1 segment of the MCA; the remaining 95 patients were excluded, as they had an occlusion of other vessels. Eighty‐five of 149 patients did not meet the eligibility criteria for this study and were therefore removed from the study population (Figure 1). Therefore, a total of 64 patients met the inclusion criteria and were enrolled in the study.

The demographic features and AIS characteristics of our study population are shown in Table S4. The mean age at AIS onset was 74.1 ± 11.9 years, and patients were similarly distributed with regard to sex. Two patients (3.1%) reported a family history of neurodegenerative disorders (both had a parent affected by Parkinson's disease). One patient (1.6%) had a personal history of exposure to DRBAs (chronic treatment of diagnosed schizophrenia).

The M1 occlusion of the MCA was localized on the right side in 33/64 patients (51.6%). The most frequent cause of AIS was cardioembolic (38/64, 59.4%), followed by stroke of undetermined etiology (14/64, 21.9%); the remaining cases were either atherothrombotic or secondary to other known causes.

A total of 61 patients (95.3%) were globally independent in activities of daily living (as defined by an mRS score ≤2 [37]) at the time of AIS, while at discharge mRS score was ≤2 in 39.1% of patients. IVT was used as a bridge to MT in 23/64 patients (35.9%). Optimal recanalization after MT, as defined by mTICI score = 3, was achieved in most patients (75%); mTICI scores 2B and 2C were obtained in 7.8% and 17.2% of patients, respectively. The mean NIHSS score at onset was 14.4 ± 6.6, while the mean NIHSS score at discharge was 5.3 ± 8.1.

Of the 64 patients, 42 underwent baseline evaluation during the acute phase of stroke, within 1 week after the event, and none of these presented a movement disorder (Table S5). The remaining 22 patients were not assessable at baseline due to severe hemiparesis, unstable clinical conditions, or limited compliance. In our study population, the mean UPDRS scores at baseline were 0.8 ± 1.1, 0.9 ± 1.3 and 4.9 ± 4.5 in the UPDRS I, UPDRS II and UPDRS III subsets, respectively (Table S5) without lateralization of motor symptoms (UPDRS III scores contralateral and homolateral to the ischemic lesion were 1.7 ± 2.0 and 1.5 ± 1.6, respectively). No patient showed RAS, while MMs were reported in six patients (14.3%).

At baseline, the MoCA scale was administered to 41/64 (64%) patients; their mean MoCA score was 12.4 ± 6.1. The remaining 23 patients were not assessable due to limited compliance.

Favorable clinical outcome, as defined by an mRS score ≤2 3 months after the AIS, was achieved in 42/64 patients (66.6%).

The 6‐ and 12‐month follow‐ups were completed by 25 and 13 patients, respectively. Loss to 6‐ and 12‐month follow‐up, either due to consent withdrawal, mRS score >4 or COVID‐19 pandemic‐related restrictions, accounted for 27 and 23 patients, respectively. The remaining patients had not yet entered either the 6‐ or 12‐month follow‐up time windows at the time of manuscript draft.

### Follow‐up assessments

The demographic features and AIS characteristics of patients who underwent the 6‐ and 12‐month follow‐up evaluations are shown in Table S4. Clinical assessments are summarized in Table S5.

**FIGURE 2:**
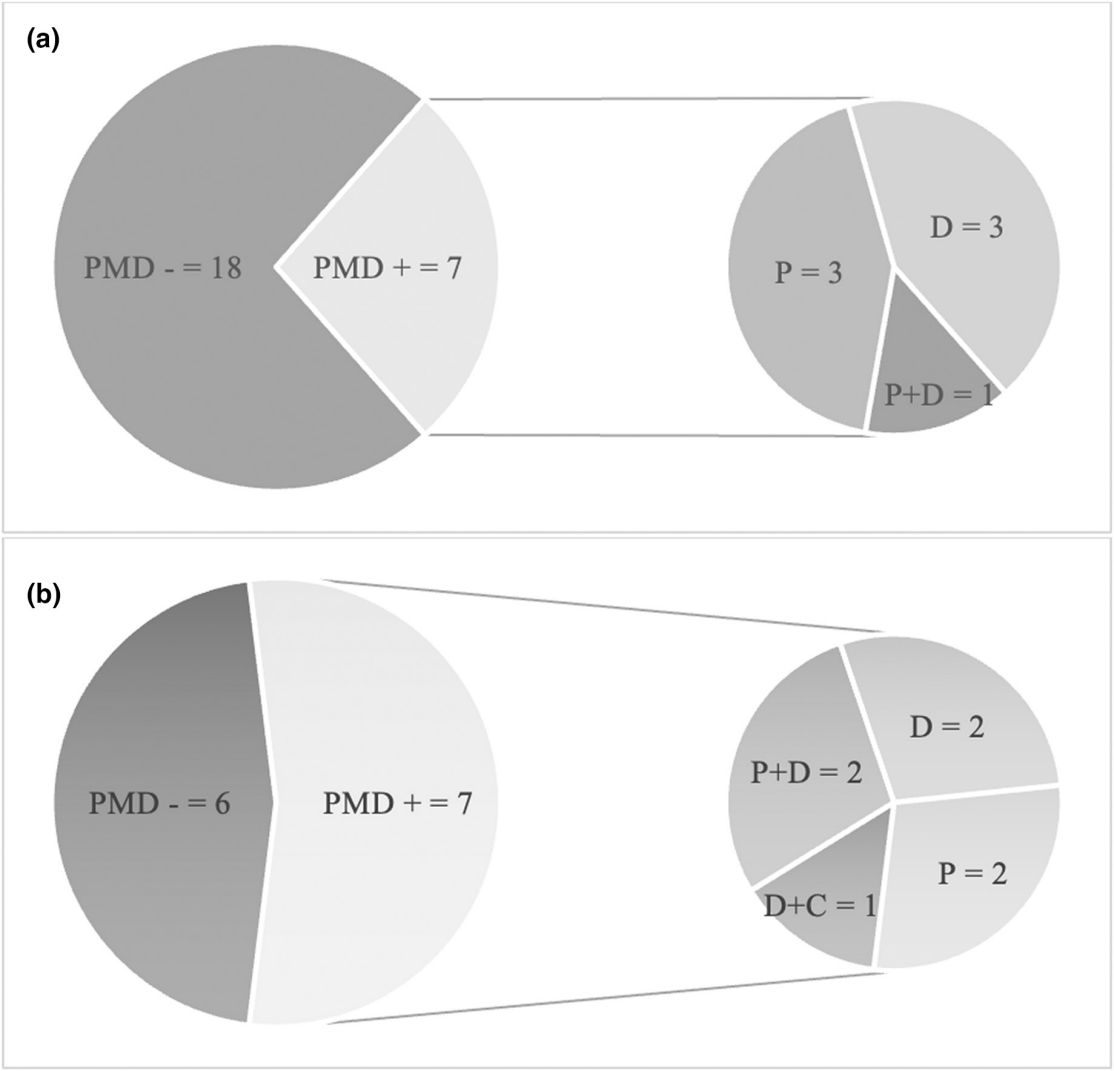
Prevalence and clinical subtype of post‐stroke movement disorders (PMDs) 6 months (a) and 12 months (b) after the acute event. D, dystonia; D+C, combined dystonia‐chorea; PMD−, patients who did not develop PMD during the follow‐up; PMD+, patients who developed a PMD throughout the follow‐up; P, parkinsonism; P+D, combined parkinsonism‐dystonia.

At 6 and 12 months after the stroke, 7/25 (28%) and 7/13 patients (53.8%), respectively, presented with an identifiable movement disorder (Figure 2).

At the 6‐month visit, three patients presented with parkinsonism, three patients with dystonia, and one patient with both parkinsonism and dystonia.

At the 12‐month follow‐up, three out of the seven patients with a movement disorder presented with a new‐onset PMD, while three patients continued showing the PMD observed at the 6‐month follow‐up evaluation. The remaining patient developed a new onset of hand and foot dystonia in combination with the parkinsonism showed at the 6‐month follow‐up visit.

Parkinsonism and dystonia were equally observed at both observational time points (three and two patients each at 6 and 12 months, respectively). In a minority of patients, we observed combined movement disorders (parkinsonism with dystonia and chorea with dystonia).

The PMDs primarily affected the body side contralateral to the brain ischemic lesion, showing a clearly asymmetric distribution in most patients (6/7 patients and 5/7 patients at 6 and 12 months, respectively). The two remaining patients developed a parkinsonian syndrome characterized by bilateral involvement and prominent axial symptoms (sum of UPDRS III 23–26 subscores ≥8): one patient developed the PMD 6 months after the AIS, and this persisted at the 12‐month follow‐up, while the second patient presented with new onset of bilateral parkinsonism after 12 months. For these patients, MRI findings revealed a right‐sided ischemic lesion involving the insula, internal capsule, caudate and putamen in one patient and a selective lesion of the left putamen in the other patient.

Dystonia was either focal (hand dystonia), segmental (bi‐brachial), multifocal (same side hand + foot dystonia) or, in one patient, hemidystonia combined with hemichorea (Table S3). The dystonic symptoms were in most cases contralateral to the AIS; similarly, in the patient presenting with bi‐brachial dystonia, the most affected body side was contralateral to the BG ischemic lesion.

At 6 and 12 months, MMs were present in 28% and 30.1% of patients, respectively, while RAS was present in 76% and 61.5% of patients. Both features were more frequently observed among patients who developed PMDs (Table 1), mostly contralaterally to the BG ischemic lesion; bilateral RAS, although more prominent contralaterally to the AIS, was found in two patients with bilateral parkinsonism and prominent axial features.

**TABLE 1:** Clinical features and assessments of patients undergoing 6‐ and 12‐month follow‐up visits.

Variable	6‐month follow‐up		*p* value	12‐month follow‐up		*p* value
	PMD− (*n*/*N* = 18/25)	PMD+ (*n*/*N* = 7/25)		PMD− (*n*/*N* = 6/13)	PMD+ (*n*/*N* = 7/13)	
Age, mean (±SD) years	71.7 (±14.6)	77.0 (±6.6)	0.7^†^	70.1 (±17.4)	78.7 (±6.2)	0.5^†^
Male sex, *n*/*N* (%)	11/18 (61.1)	2/7 (28.6)	1^^^	3/6 (50.0)	2/7 (28.6)	0.6^§^
Family history, *n*/*N* (%)	2/18 (11.1)	0/7 (0)	1^^^	0/6 (0)	1/7 (14.3)	0.5^§^
History of DRBA exposure, *n*/*N* (%)	1/18 (0.6)	0/7 (0)	1^^^	0/6 (0)	0/7 (0)	–
Comorbidities, *n*/*N* (%)			0.5^^^			
Previous ischemic stroke	1/18 (5.5)	0/7 (0)	–	1/6 (16.7)	0/7 (0)	0.5^§^
Diabetes	0/18 (0)	0/7 (0)	0.3^^^	0/6 (0)	0/7 (0)	–
Hypertension	12/18 (66.7)	6/7 (85.7)	0.8^^^	5/6 (83.3)	4/7 (57.1)	0.6^§^
Dyslipidemia	6/18 (33.3)	2/7 (28.6)	1^^^	4/6 (66.7)	2/7 (28.6)	0.3^§^
Active smoker	5/18 (27.8)	2/7 (28.6)		2/6 (33.3)	2/7 (28.6)	1^§^
NIHSS score at onset, mean (±SD)	11.6 (±7.7)	15.6 (±6.6)	0.6^†^	13.5 (±8.8)	10.1 (±5.4)	0.2^†^
NIHSS score at discharge, mean (±SD)	2.1 (±2.2)	3.9 (±2.6)	0.1^†^	0.7 (±1.0)	4.0 (±3.3)	**0.047** ^†^
mTICI score			0.6^^^			0.2^^^
2B, *n*/*N* (%)	1/18 (5.6)	1/7 (14.3)		2/6 (33.3)	2/7 (28.6)	
2C, *n*/*N* (%)	3/18 (16.7)	2/7 (28.6)		1/6 (16.7)	2/7 (28.6)	
3, *n*/*N* (%)	14/18 (77.7)	4/7 (57.1)		2/6 (33.3)	3/7 (42.8)	
Pre‐event mRS score = 0, *n*/*N* (%)	14/18 (77.7)	5/7 (71.4)	0.4^^^	6/6 (100)	6/7 (85.7)	0.3^^^
mRS score at discharge ≤2, *n*/*N* (%)	14/18 (77.7)	2/7 (28.6)	0.07^^^	6/6 (100)	4/7 (57.1)	0.2^^^
mRS score at 3 months ≤2, *n*/*N* (%)	17/18 (94.4)	3/7 (42.9)	**0.01** ^^^	6/6 (100)	4/7 (57.1)	0.2^^^
Intravenous thrombolysis, *n*/*N* (%)	8/18 (44.4)	1/7 (14.3)	0.3^^^	4/6 (66.7)	2/7 (28.6)	0.3^§^
Right side stroke, *n*/*N* (%)	11/18 (61.1)	3/7 (42.9)	0.7^^^	2/6 (33.3)	5/7 (71.4)	0.3^§^
UPDRS I total score, mean (±SD)	2.5 (±2.1)	3.0 (±1.9)	0.6^†^	1.7 (±1.9)	3.4 (±2.6)	0.2^†^
UPDRS II total score, mean (±SD)	5.1 (±6.6)	9.2 (±5.2)	**0.02** ^†^	2.8 (±2.3)	10.7 (±7.8)	0.05^†^
UPDRS III score						
Total, mean (±SD)	9.5 (±5.0)	16.9 (±8.4)	0.06^†^	8.3 (±3.1)	25.0 (±13.1)	**0.01** ^†^
Axial, mean (±SD)	2.0 (±1.6)	4.4 (±3.6)	0.1^†^	0.8 (±1.3)	7.0 (±3.9)	**<0.01** ^†^
Contralateral, mean (±SD)	3.4 (±1.8)	5.0 (±1.8)	0.1^†^	3.7 (±2.2)	8.1 (±3.2)	**0.03** ^†^
Homolateral, mean (±SD)	2.1 (±2.4)	3.6 (±2.3)	0.6^†^	2.2 (±1.5)	5.1 (±4.7)	0.2^†^
RAS opposite to lesion, *n*/*N* (%)	12/18 (66.6%)	7/7 (100%)	0.2^^^	2/6 (33.3%)	6/7 (85.7%)	0.05^^^
MoCA score, mean (±SD)	19.5 (±5.6)	14.7 (±5.4)	0.06^†^	22.0 (±5.2)	21.5 (±3.4)	1^†^
MMs, *n*/*N* (%)	4/18 (22.2%)	3/7 (42.9%)	0.6^^^	0/6 (0%)	4/7 (57.1%)	0.1^^^

Abbreviations: DRBA, dopamine receptor blocking agent; MM, mirror movement; MoCA, Montreal Cognitive Assessment; mRS, modified Rankin scale; mTICI: modified treatment in cerebral infarction; NIHSS, National Institutes of Health Stroke Scale; PMD−, patients who did not develop post‐stroke movement disorders during the follow‐up; PMD+, subjects who developed a post‐stroke movement disorder throughout the follow‐up; RAS, reduced arm swing; UPDRS, Unified Parkinson's Disease Rating Scale.

Bold values correspond to significant results.

^†^ Mann–Whitney *U*‐test.

^§^ Fisher's exact test.

^^^ Pearson's chi‐squared test with Yates' correction.

The mean MoCA score was significantly lower in the acute phase (12.4 ± 6.1) than at the 6‐month (18.1 ± 5.5; *p* = 0.008) and 12‐month visit (21.8 ± 4.3; *p* = 0.03), as shown in Table S5. Comparisons of the clinical and demographic features between the groups of patients who have and have not developed a PMD throughout the follow‐up are shown in Table 1.

The subgroup of patients who developed a PMD at 6 months showed significantly higher scores on the UPDRS II (*p* = 0.02) and worse global clinical outcome, assessed according to mRS score 3 months after the AIS (*p* = 0.01).

The subset of patients who developed a PMD 1 year after the AIS showed greater motor impairment in the body side opposite to the ischemic lesion (*p* = 0.03) and more severe axial symptoms (*p* < 0.01). In our cohort, NIHSS scores at the time of discharge were significantly higher in patients who subsequently developed a PMD (*p* = 0.047).

The within‐group comparison of clinical features assessed at baseline and at the follow‐up visits showed a significant increase of UPDRS I and MoCA scores and of the frequency of RAS contralateral to the lesion in patients who did not develop PMDs at 6 months. Conversely, no statistically significant differences were found for patients who developed new‐onset PMDs (Table S1). No significant differences were found in the within‐group comparison of the clinical features assessed at baseline and at the 12‐month follow‐up (Table S6).

Of the conditions evaluated, namely, diabetes, hypertension, dyslipidemia, previous ischemic stroke and tobacco smoking, none of the known risk factors for AIS (Table 1), nor any AIS etiological mechanism were specifically associated with the development of PMDs (Table S2).

### Neuroradiological findings

**FIGURE 3:**
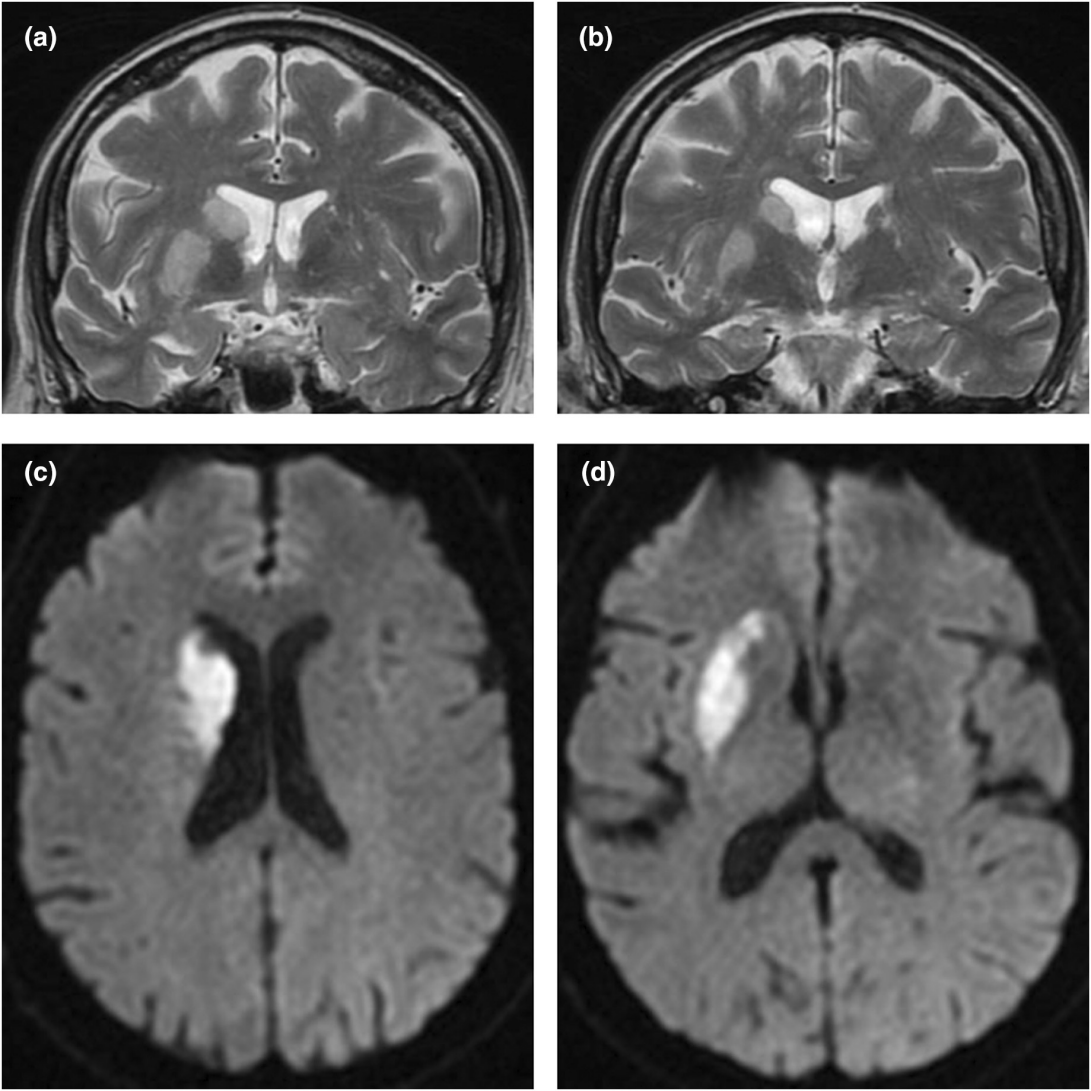
Selective ischemic lesion of the basal ganglia occurring after an acute proximal occlusion of the middle cerebral artery in a patient successfully treated with mechanical thrombectomy from our cohort. Panels a and b: T2‐weighted sequences coronal sections at the level of the basal ganglia. Panels c and d panels: diffusion‐weighted sequences axial sections at the level of the basal ganglia.

In our population, the putamen was the most frequent site of ischemic damage, with this being affected in up to 81.2% of patients (Figure 3). The AIS resulted in selective involvement of a single subcortical structure in five patients (7.8%). Twelve patients (18.7%) showed a selective ischemic lesion of the BG, with relative sparing of the internal capsule and cortical structures.

Similar neuroradiological findings at baseline were observed for both the 6‐ and 12‐month follow‐up population subgroups: the most affected subcortical structure was the putamen, which was involved in 20/25 (80%) and 10/13 patients (76.9%), respectively; a selective lesion of the BG was found in 6/25 (24%) and 2/13 patients (15.4%) at 6 and 12 months, respectively. Finally, a selective involvement of a single subcortical structure was found in 2/25 patients (8%) at the 6‐month follow‐up and in none of the patients undergoing the 12‐month follow‐up.

No correlation was found between the structure affected by the AIS and the subsequent development of PMDs at 6 and 12 months (Table 2).

**TABLE 2:** Subcortical structures affected by the acute ischemic stroke in patients with and without delayed onset of post‐stroke movement disorder at 6‐ and 12‐month follow‐ups.

Site of lesion	6‐month follow‐up		*p* value	12‐month follow‐up		*p* value
	PMD− *n*/*N*, %	PMD+ *n*/*N*, %		PMD− *n*/*N*, %	PMD+ *n*/*N*, %	
IC	3/18, 16.7%	5/7, 71.4%	0.06	1/6, 16.7%	5/7, 71.4%	0.1
Ca	9/18, 50%	3/7, 42.8%	1	3/6, 50%	2/7, 28.6%	0.6
Pu	13/18, 72.2%	7/7, 100%	0.3	4/6, 66.6%	6/7, 85.7%	0.6
Tha	2/18, 11.1%	2/7, 28.6%	0.5	0/6, 0%	3/7, 42.8%	0.2
GP	7/18, 38.9%	4/7, 57.1%	0.7	3/6, 50%	4/7, 57.1%	1
Ins	8/18, 44.4%	3/7, 42.8%	1	3/6, 50%	3/7, 42.9%	1
OA	11/18, 61.1%	6/7, 85.7%	0.4	3/6, 50%	7/7, 100%	0.07

*Note*: Comparisons were carried out with Fisher's exact test.

Abbreviations: Ca, caudate; GP, globus pallidus; IC, internal capsule; Ins, insula; OA, other areas; PMD−, subjects who did not develop post‐stroke movement disorders during the follow‐up; PMD+, subjects who developed a post‐stroke movement disorder throughout the follow‐up; Pu, Putamen; Tha, thalamus.

## DISCUSSION

In this study, we aimed to investigate the frequency and clinical features of movement disorders occurring after an AIS due to a proximal MCA occlusion successfully treated with MT. Such ischemic lesions can present as a model of relatively selective damage of the BG. Effective MT normally cannot avert the lenticulate infarction, regardless of the recanalization achieved [17], not infrequently producing an isolated ischemic lesion of the BG [16].

In our cohort, a variety of PMDs was developed throughout the follow‐up period, ranging from isolated parkinsonism or dystonia to combined movement disorders (dystonia‐parkinsonism or chorea‐dystonia), supporting the high clinical heterogeneity of these disorders. Parkinsonism and dystonia were the most frequent PMDs, and were approximately equally distributed in our population.

Among those assessed at baseline, no patient developed a movement disorder acutely after the AIS. PMDs occurring shortly after the AIS are usually hyperkinetic and are often secondary to cortical or posterior cerebral circulation infarcts (e.g., subthalamic nucleus, posterior thalamus) [1, 10, 11, 38, 39]; the relative sparing of such structures in our population, owing to the effective recanalization leading to a selective subcortical involvement and the exclusion of AIS due to posterior circulation LVO, might explain the absence of acute PMDs observed in our cohort. We observed a delayed onset of movement disorders in a significant proportion of patients, even later than 6 months after the event. Interestingly, the prevalence of PMDs seemed to increase when extending the observational period from 6 to 12 months, although this finding should be interpreted with caution because of the sample size. Such a trend is in contrast to the usual recovery process from an AIS, in which the patient generally improves with time. This finding may be explained by the intrinsic nature of network impairment in PMDs [1]. In fact, PMDs may arise not only due to the tissue loss, but also as a result of maladaptive neuroplasticity and aberrant compensatory circuitry originating after ischemic injury [3, 4, 40, 41]. Accordingly, in our study motor impairment seemed to be greater at a longer time after the ischemic event. Indeed, at the 12‐month follow‐up, patients with PMDs had significantly higher UPDRS III subscores, with more severe axial and contralateral symptoms.

In our cohort, the PMDs primarily affected the side contralateral to the ischemic lesion in most patients, in line with previous descriptions in the literature [1, 3]. Nonetheless, two patients developed bilateral parkinsonism with prominent axial features in the long‐term follow‐up. Both patients had an ischemic lesion of the putamen, which, in one patient, was selectively involved; this finding is in line with the current knowledge of bilateral somatic control of nigrostriatal pathways [42].

The overall prevalence of MMs and RAS was remarkable in our population and increased over time; conversely, these clinical features were not evident in any patient acutely after the ischemic event, suggesting a time‐dependent pathological mechanism. RAS was found during follow‐up even in a remarkable proportion of patients without overt PMDs. Although such clinical features are sometimes present in healthy patients [43], in patients affected by BG ischemic lesions they might represent early signs of a movement disorder, as for RAS in Parkinson's disease [44, 45], and warrant a longer follow‐up.

The brain structure most frequently affected by the ischemic lesion was the putamen in our cohort (Figure S1). Moreover, in line with previously published studies [16, 18], in almost 20% of patients the neuroradiological assessment highlighted selective involvement of the BG following the AIS, with relative sparing of the lobar cortices and deep white matter structures such as the internal capsule.

Neither the clinical characteristics at baseline nor the site of the ischemic lesion significantly correlated with the development of PMDs, possibly due to the limited sample size. A possible link between PMD phenomenology and ischemic lesion site was recently proposed using a functional network‐based rather than a structural approach to stroke localization [5, 46], which may constitute a promising future study direction. The higher NIHSS score at discharge along with greater 3‐month mRS and 6‐month UPDRS II scores in the group who developed a PMD may suggest that patients with more clinically severe AIS are at higher risk for developing PMDs, although such results need to be confirmed in a larger population.

Throughout the observational period, cognitive performance assessed with the MoCA test seemed to improve in all groups, as expected, although statistical significance was not achieved for all comparisons. These findings confirm the recently described relevant impact of post‐thrombectomy BG selective infarction on cognition [47].

Up to more than 50% (7/13) of the patients evaluated 1 year after the AIS in this study population showed findings of PMDs; since previous retrospective studies observed a prevalence of 1%–4% [7, 9], we suggest that PMDs may be underdiagnosed, particularly in patients with selective involvement of the BG. Currently, even in tertiary centers, there is often no specific competent clinic for the systematic evaluation and treatment of patients presenting with such pathology. Given the typical delayed onset and clinical heterogeneity of PMDs, there is currently an unmet need for specialist long‐term follow‐up in patients developing ischemic lesions of the BG after MT to prevent the underdiagnosis and undertreatment of these disorders.

The main limitations of this study were the sample size, as several patients were lost to follow‐up, and the lack of a control group. It is reasonable to expect more robust results with larger studies and longer observational periods; therefore, it is essential to study this relatively new lesion‐induced movement disorders model extensively.

In conclusion, PMDs occurring after ischemic injury of the BG are not uncommon at long‐term follow‐up and might be underrecognized. The clinical relevance of this phenomenon might grow in the future, due to the expanding indications for MT in the treatment of AIS and the dramatic increase of stroke survivors with excellent functional recovery. These disorders are characterized by a delayed onset and clinical heterogeneity, requiring a prolonged and specialized medical observation after the acute ischemic event to be intercepted and managed.

## AUTHOR CONTRIBUTIONS

Study concept and design: Giacomo Della Marca, Carla Piano, Valerio Brunetti, Valeria Guglielmi and Paolo Calabresi. Data collection and interpretation: Leonardo Rigon, Danilo Genovese, Angelo Tiziano Cimmino, Irene Scala, Salvatore Citro, Giovanna Masone, Eleonora Rollo, Riccardo Di Iorio, Mauro Monforte, Roberta Morosetti and Pietro Caliandro. Drafting of the manuscript: Leonardo Rigon, Danilo Genovese, Giacomo Della Marca, Valeria Guglielmi and Valerio Brunetti. Critical revision of the manuscript: Giacomo Della Marca, Paolo Calabresi, Carla Piano, Anna Rita Bentivoglio, Valerio Brunetti, Valeria Guglielmi, Alessandro Pedicelli, Anselmo Caricato, Aldobrando Broccolini and Giovanni Frisullo.

## CONFLICT OF INTEREST STATEMENT

The authors declare no competing interests.

## Supporting information


Appendix S1:

## Data Availability

The data that support the findings of this study are available from the corresponding author upon reasonable request.
