# Supplementary material for: Movement disorders following mechanical thrombectomy resulting in ischemic lesions of the basal ganglia: An emerging clinical entity
Source: Eur J Neurol. 2024 Feb 1;31(5):e16219. doi: 10.1111/ene.16219 (PMC11235728; doi:10.1111/ene.16219)
Supplement: Supplementary file 1 — Appendix S1: [file ENE-31-e16219-s001.docx]

Supplementary information online

Supplementary Table 1: Progression of clinical features at the 6-months follow-up.

| Variable, mean (± SD) | PMD- (n=18) | | *p-value* | PMD+ (n=7) | | *p-value* |
| --- | --- | --- | --- | --- | --- | --- |
|  | **Baseline** | **6 months** |  | **Baseline** | **6 months** |  |
| UPDRS I total | 0.2 (± 0.5) | 2.5 (± 2.1) | ***0.03****^†^* | 1.3 (± 1.5) | 3.0 (± 1.9) | *0.5****^†^*** |
| UPDRS II total | 1.1 (± 1.5) | 5.1 (± 6.6) | *0.09****^†^*** | 1 (± 1.4) | 9.2 (± 5.2) | *1****^†^*** |
| UPDRS III |  |  |  |  |  |  |
| total | 6.5 (± 4.7) | 9.5 (± 5.0) | *0.4****^†^*** | 10.3 (± 2.5) | 16.9 (± 8.4) | *0.2****^†^*** |
| axial | 0.8 (± 1.4) | 2.0 (± 1.6) | *0.1****^†^*** | 1.3 (± 2.3) | 4.4 (± 3.6) | *0.5****^†^*** |
| contralateral to AIS | 2.3 (± 1.6) | 3.4 (± 1.5) | *0.3****^†^*** | 4.3 (± 0.6) | 5.0 (± 2.1) | *0.2****^†^*** |
| homolateral to AIS | 2 (±1.9) | 2.1 (± 1.4) | *0.8****^†^*** | 2.3 (± 1.5) | 3.6 (± 3.7) | *0.4****^†^*** |
| RAS opposite to AIS, n/N (%) | 0/12 (0%) | 12/18 (66.6%) | ***0.001^§^*** | 0/3 (0%) | 7/7 (100%) | *0.3^§^* |
| MoCA | 14.3 (± 7.0) | 19.5 (± 5.1) | ***0.02^†^*** | 10.2 (± 5.3) | 14.7 (± 5.3) | *0.3****^†^*** |
| Mirror movements, n/N (%) | 3/12 (25.0%) | 4/18 (22.2%) | *0.8^§^* | 0/3 (0%) | 3/7 (42.9%) | *0.5^§^* |

Abbreviations: PMD-: subjects who did not develop post-stroke movement disorders during the follow-up; PMD+: subjects who developed a post-stroke movement disorder throughout the follow-up; UPDRS: Unified Parkinson’s Disease Rating Scale; RAS: reduced arm swing; MoCA: Montreal Cognitive Assessment.

^†^ Wilcoxon-Mann–Whitney test; ^§^ Pearson’s chi squared test with Yates’ correction.

Supplementary Table 2. Acute ischemic stroke etiologic subtype according to the TOAST classification in patients with and without delayed onset of post-stroke movement disorders at 6- and 12-months follow-up.

| TOAST subtype | 6-month follow-up | | *p-value* | 12-month follow-up | | *p-value* |
| --- | --- | --- | --- | --- | --- | --- |
|  | **PMD-** n/N, % | **PMD+** n/N, % |  | **PMD-** n/N, % | **PMD+** n/N, % |  |
| Atherothrombotic | 0/18, 0% | 0/7, 0% | *--* | 0/6, 0% | 1/7, 14.3% | *1* |
| Cardioembolic | 12/18, 66.7% | 3/7, 42.8% | *0.4* | 5/6, 83.3% | 2/7, 28.6% | *0.1* |
| Small vessels disease | 0/18, 0% | 0/7, 0% | *--* | 0/6, 0% | 0/7, 0% | *--* |
| Other known cause | 2/18, 11.1% | 1/7, 14.3% | *1* | 0/6, 0% | 0/7, 0% | *--* |
| Undetermined etiology | 4/18, 22.2% | 3/7, 42.8% | *0.3* | 1/6, 16.7% | 4/7, 57.1% | *0.3* |

Abbreviations: TOAST^29^ etiologic classification of the AIS. PMD-: subjects who did not develop post-stroke movement disorders during the follow-up; PMD+: subjects who developed a post-stroke movement disorder throughout the follow-up.

Comparisons were carried out with Fischer’s exact test.

Supplementary Table 3. Clinical features at different timepoints of patients presenting with dystonic symptoms.

| **Patient** | **Baseline (n=42)** | | | **6-months FU (n=25)** | | | **12-months FU (n=13)** | | |
| --- | --- | --- | --- | --- | --- | --- | --- | --- | --- |
|  | **Distribution** | **BFMDRS** | **MM** | **Distribution** | **BFMDRS** | **MM** | **Distribution** | **BFMDRS** | **MM** |
| **Pt 1** | No dystonia | -- | No | multifocal | 6/120 | + | NA | NA | NA |
| **Pt 2** | No dystonia | -- | No | NA | NA | NA | hemidystonia | 18/120 | + |
| **Pt 3** | No dystonia | -- | No | multifocal | 12/120 | No | multifocal | 11/120 | + |
| **Pt 4** | No dystonia | -- | No | focal | 4/120 | + | Focal | 4/120 | No |
| **Pt 5** | No dystonia | -- | No | No dystonia | -- | No | multifocal | 16/120 | + |
| **Pt 6** | No dystonia | -- | No | Focal | 3/120 | + | Focal | 3/120 | + |

Abbreviations: FU: follow-up; BFMDRS: Burke-Fahn-Marsden Dystonia Rating Scale; MM: Mirror movements; NA assessment not performed.

Supplementary Table 4: Study population demographic features and acute ischemic stroke characteristics.

| **Variable** | **Study population (n=64)** | **6-month follow-up subgroup (n=25)** | **12-month follow-up subgroup (n=13)** |
| --- | --- | --- | --- |
| Age, mean (± SD)  Male sex, n/N (%)  Family history, n/N (%) | 74.08 (± 11.9)  27/64 (42.2%) | 73.20 (± 12.9)  13/25 (52.0%) | 74.77 (± 12.8)  5/13 (38.5%) |
|  | 2/64 (3.1%) | 2/25 (8.0%) | 1/13 (7.7%) |
| History of exposure to DRBA, n/N (%)  Right side stroke, n/N (%)  Comorbidities, n/N (%)  Previous ischemic stroke  Diabetes  Hypertension  Dyslipidemia  Active smoker | 1/64 (1.6%)  33/64 (51.6%) | 1/25 (4.0%)  14/25 (56%) | 0/13 (0%)  7/13 (53.8%) |
|  |  |  |  |
|  | 5/64 (7.8%)  7/64 (10.9%)  51/64 (79.7%)  22/64 (34.4%)  13/64 (20.3%) | 1/25 (4.0%)  0/25 (0%)  18/25 (72%)  8/25 (32%)  7/25 (28%) | 1/13 (7.7%)  0/13 (0%)  9/13 (69.2%)  6/13 (46.1%)  4/13 (30.8%) |
| NIHSS at onset, mean (± SD)  NIHSS at discharge, mean (± SD)  mTICI score  2B, n/N (%)  2C, n/N (%)  3, n/N (%)  Pre-event mRS,  ≤2, n/N (%)  >2, n/N (%)  mRS at discharge,  ≤2, n/N (%)  >2, n/N (%)  mRS at 3 months,  ≤2, n/N (%)  >2, n/N (%)  unknown, n/N (%)  Intravenous thrombolysis, n/N (%)  TOAST classification of stroke, n/N (%)  Type 1 (atherothrombotic)  Type 2 (cardioembolic)  Type 3 (small vessels)  Type 4 (other known cause)  Type 5 (undetermined etiology) | 14.4 (± 6.6) | 12.7 (± 7.5) | 11.7 (± 7.1) |
|  | 5.3 (± 8.1) | 2.6 (± 2.4) | 2.5 (± 3.0) |
|  |  |  |  |
|  | 5/64 (7.8%) | 2/25 (8.0%) | 4/13 (30.8%) |
|  | 11/64 (17.2%) | 4/25 (16.0%) | 2/13 (15.4%) |
|  | 48/64 (75.0%) | 29/25 (76.0%) | 7/13 (53.8%) |
|  | 61/64 (95.3%)  3/64 (4.7%)  25/64 (39.1%)  39/64 (60.9%)  30/64 (46.9%)  15/64 (23.4%)  19/64 (32.8%)^†^  23/64 (35.9%)  6/64 (9.4%)  38/64 (59.4%)  0/64 (0%)  6/64 (9.4%)  14/64 (21.8%) |  |  |
|  |  | 24/25 (96.0%) | 13/13 (100%) |
|  |  | 1/25 (4.0%) | 0/13 (0%) |
|  |  |  |  |
|  |  | 16/25 (64.0%) | 10/13 (76.9%) |
|  |  | 8/25 (32.0%) | 2/13 (15.4%) |
|  |  |  |  |
|  |  | 19/25 (76.0%) | 10/13 (76.9%) |
|  |  | 6/25 (24.0%) | 3/13 (13.1%) |
|  |  | - | - |
|  |  | 9/25 (37.5%)  0/25 (0%)  15/25 (60.0%)  0/25 (0%)  3/25 (12.0%)  7/25 (28.0%) | 6/13 (46.1%)  1/13 (7.7%)  7/13 (53.8%)  0/13 (0%)  0/13 (0%)  5/13 (38.5%) |

Abbreviations: mTICI: modified treatment in cerebral infarction; mRS: modified Rankin scale; TOAST: Trial of Org 10172 in Acute Stroke Treatment; NIHSS: National Institutes of Health Stroke Scale; DRBA: dopamine receptor blocking agents; MoCA: Montreal Cognitive Assessment. ^†^Incomplete follow-up by the time of manuscript draft (less than 3 months since the acute event).

Supplementary Table 5: Clinical features at baseline, 6- and 12-months follow-up

| **Variable** | **Baseline (n=42)** | **6-month FU (n=25)** | **12-month FU (n=13)** |
| --- | --- | --- | --- |
| **UPDRS I total, mean (**± **SD)** | 0.8 (± 1.1) | 0.5 (±0.8) | 0.5 (± 1.1) |
| **UPDRS II total, mean (**± **SD)** | 0.9 (±1.3) | 1.1 (± 1.4) | 0.5 (± 0.5) |
| **UPDRS III** |  |  |  |
| **total, mean (**± **SD)** | 4.9 (±4.5) | 7.3 (± 4.5) | 6.6 (± 3.6) |
| **axial, mean (**± **SD)** | 0.4 (± 1.0) | 0.9 (± 1.5) | 0.6 (± 1.1) |
| **contralateral to AIS, mean (**± **SD)** | 1.7 (±2.0) | 2.7 (± 1.7) | 2.6 (± 1.7) |
| **homolateral to AIS, mean (**± **SD)** | 1.5 (± 1.6) | 2.1 (± 1.8) | 2.1 (± 1.5) |
| **RAS opposite to AIS, n/N (%)** | 0/42 (0%) | 19/25 (76%) | 8/13 (61.5%) |
| **Mirror movements, n/N (%)** | 6/42 (14.3%) | 7/25 (28%) | 4/13 (30.8%) |
| **MoCA, mean (**± **SD)** | 12.4 (± 6.1) | 18.1 (± 5.5) | 21.8 (± 4.3) |

Abbreviations: FU: follow-up; UPDRS: Unified Parkinson’s Disease Rating Scale; RAS: reduced arm swing; MoCA: Montreal Cognitive Assessment

Supplementary Table 6: Progression of clinical features at the 12-months follow-up.

| Variable, mean (± SD) | PMD- (n=6) | | *p-value* | PMD+ (n=7) | | *p-value* |
| --- | --- | --- | --- | --- | --- | --- |
|  | **Baseline** | **12 months** |  | **Baseline** | **12 months** |  |
| UPDRS I total | 0.2 (± 0.5) | 1.6 (± 1.7) | *0.2^†^* | 1.0 (± 1.7) | 3.8 (± 2.6) | *0.4****^†^*** |
| UPDRS II total | 0.7 (± 0.5) | 3.6 (± 2.9) | *0.1****^†^*** | 0 (± 0) | 10.8 (± 8.5) | *--****^†^*** |
| UPDRS III |  |  |  |  |  |  |
| total | 7.0 (± 2.9) | 11.1 (± 8.0) | *0.2****^†^*** | 5.5 (± 6.4) | 24.8 (± 14.3) | *0.5****^†^*** |
| axial | 0.6 (± 1.3) | 2.1 (± 3.6) | *--****^†^*** | 0.5 (± 0.7) | 6.5 (± 4.0) | *0.5****^†^*** |
| contralateral to AIS | 2.6 (± 1.1) | 4.4 (± 3.2) | *0.1****^†^*** | 2.5 (± 3.5) | 8.3 (± 3.5) | *0.5****^†^*** |
| homolateral to AIS | 2.2 (±1.1) | 2.4 (± 3.7) | *1****^†^*** | 2.0 (± 2.8) | 5.3 (± 5.1) | *1****^†^*** |
| RAS opposite to AIS | 0/5 (0%) | 2/6 (33.3%) | *0.5^§^* | 0/2 (0%) | 6/7 (83.3%) | *0.2^§^* |
| MoCA | 7.8 (± 8.3) | 22.0 (± 4.7) | *0.1^†^* | 13.8 (± 4.3) | 21.5 (± 4.4) | *0.2****^†^*** |
| Mirror movements, n/N (%) | 0/5 (0%) | 0/6 (%) | *--* | 0/2 (0%) | 4/7 (57.1%) | *0.5^§^* |

Abbreviations: PMD-: subjects who did not develop post-stroke movement disorders during the follow-up; PMD+: subjects who developed a post-stroke movement disorder throughout the follow-up; UPDRS: Unified Parkinson’s Disease Rating Scale; RAS: reduced arm swing; MoCA: Montreal Cognitive Assessment.

† Wilcoxon-signed rank test; ^§^ Pearson’s chi squared test with Yates’ correction.


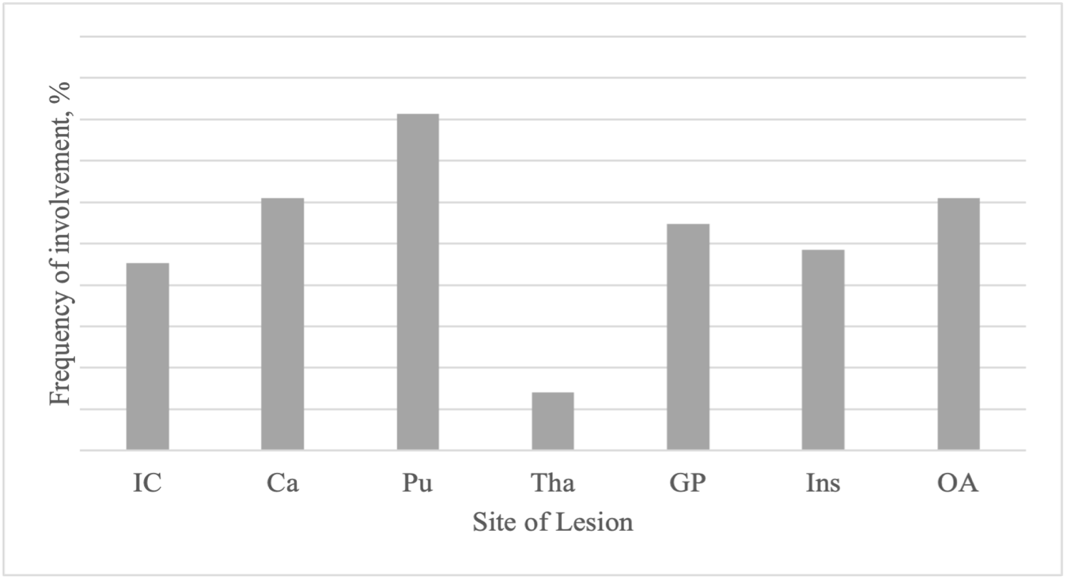


Supplementary Figure 1 Frequency of ischemic involvement of the cerebral sites in the study population;
Abbreviations: IC: internal capsule; Ca: caudate; Pu: putamen; Tha: thalamus; GP: globus pallidus; Ins: insula; OA: other areas
